# Psoriasis vulgaris and familial cancer risk- a population-based study

**DOI:** 10.1186/1897-4287-11-6

**Published:** 2013-06-28

**Authors:** Romuald Maleszka, Katarzyna Paszkowska-Szczur, Ewa Soczawa, Magdalena Boer, Monika Różewicka-Czabańska, Joanna Wiśniewska, Aneta Mirecka, Lidia Krysztoforska, Zygmunt Adamski, Jan Lubinski, Tadeusz Dębniak

**Affiliations:** 1 Department of Dermatology and Venerology, Pomeranian Medical University, Szczecin, Poland; 2 Department of Genetics and Pathology, International Hereditary Cancer Center, Pomeranian Medical University, Połabska 4, 70-115 Szczecin, Poland; 3 Regional Hospital, Koszalin, Poland; 4 Clinic of Dermatology and Venerology, Poznan University of Medical Science, Szczecin, Poland

**Keywords:** Psoriasis vulgaris, Familial cancer risk

## Abstract

**Background:**

Follow-up studies of psoriasis patients indicate an increased risk in the occurrence of malignancies at different sites of origin. Population stratification and/or complicated interpretation of evidence on the risk of cancer (due to the small number of patients included in most series) lead to inconsistent data. Herein we investigated the risk of occurrence of malignancies at different sites of origin in a series of 517 psoriasis patients and their 1st degree relatives.

**Methods:**

We evaluated the tumour spectrum as well as the age of the patient at diagnosis of cancers in psoriasis families along with the observed and expected frequencies of malignancies. The distribution of 17 common mutations/polymorphisms in 10 known cancer susceptibility genes among psoriasis patients and 517 matched healthy controls were examined. No such study has been published to date.

**Results:**

The statistical comparison of the observed and expected frequencies of cancers revealed a higher than expected occurrence of Hodgkin’s lymphoma among males in psoriasis families when compared to the general population (OR=1.8, 95%CI 1.6-2.1, p=0.002). There was a non-significant tendency towards a younger age of onset and overrepresentation of laryngeal cancer and leukaemia in psoriasis families. We found no major differences in the distribution of cancer susceptibility mutations among our cases and the healthy controls.

**Conclusions:**

The results of our study suggest an increased risk of Hodgkin’s lymphoma for male members of psoriasis families. Further studies are needed to confirm the findings and to evaluate whether or not the application of cancer surveillance protocols for Hodgkin’s lymphoma, leukaemia and laryngeal cancer are justified in these families.

## Background

Psoriasis is one of the most common skin disorders, and it is estimated that it affects 2-3% of the general Caucasian population [1]. Psoriasis is a chronic inflammatory skin disorder characterised by keratinocyte hyperproliferation and increased cutaneous blood flow induced via stimulation of tissue resident immune cells and a marked alteration of cytokine profiles [2]. Current evidence suggests that psoriasis is an immune-mediated disorder, and innovative therapies involved in the suppression of immune responses, such as T-cell-targeted agents and tumour necrosis factor (TNF) inhibitors, have improved the outcome of the disease [3,4]. As judged by twin- and large population-based genetic studies, the disease seems to have a strong genetic component – disease concordance in monozygotic twins is at most 70%, and the sibling recurrence risk of PsV has been estimated to range between 4 and 11 [5]. As of the publication of this writing, the molecular background of this disease remains unclear [6].

At present it is generally accepted that the disease is both multifactorial and genetically heterogeneous. GWAS studies identified several loci that are associated with psoriasis [7,8]. Recently 15 new susceptibility loci (which included candidate genes whose products are involved in innate host defense) have been revealed, increasing to 36 the number associated with psoriasis in European individuals [9].

Follow-up studies of psoriasis patients point to an increased risk in the occurrence of malignancies at different sites of origin. Some reports suggest more common development of non-melanoma skin cancer among psoriasis patients [10-13]. Results of other studies point to the increased frequency of other neoplasms, such as cancers of the larynx, lung, colon, kidney, pancreas and non-Hodgkin's lymphoma [12,14-16]. Unfortunately, data from literature is not entirely consistent. According to Alderson and Clark, cancers of the skin, stomach or lung are not associated with psoriasis [17]. In a recent study of Finnish psoriasis patients, the estimated relative risk was higher for Hodgkin’s and non-Hodgkin’s lymphomas, squamous skin cancer and laryngeal cancers; the risk for other malignancies (such as colon, lung or kidney) was unchanged when compared to the general population [18].

Exposure to UVA and UVB (used in the treatment of psoriasis) most likely increases the risk of malignant melanoma [19,20]. According to Lee et al., melanoma and lymphoma are overrepresented among severe psoriasis patients [21]; however, the risk of malignant melanoma was reported to be diminished among psoriasis patients in other studies [15,18].

Inconsistent data from literature can occur due to population stratification and/or complicated interpretation of evidence on the risk of cancer due to the small number of patients included in most series, precluding a full evaluation of rare neoplasms. Furthermore, the selection of patients in a clinical series might result in assessment of the effect of special types of therapies used in subsets of patients.

Herein we have investigated the risk of occurrence of malignancies of a different site of origin in a series of 517 psoriasis patients and their 1st degree relatives. We evaluated the tumour spectrum and age of diagnosis of cancers diagnosed in PsV families, as well as the observed and expected frequencies of malignancies.

Mutations/polymorphisms of many cancer susceptibility genes (such as BRCA1, CDKN2A, XPD, VDR, etc.) lead to tumour development. Some of these “cancer risk” gene alterations were also reported to be associated with psoriasis risk [22-24]. To evaluate the possible link between psoriasis and cancer, we examined the distribution of 17 mutations/polymorphisms in 10 known cancer susceptibility genes among patients and 517 matched healthy controls.

No such study has been published to date.

### Patients and methods

#### Patients

The case group consisted of 517 (218 women and 299 men, mean age 42.9) unselected, consecutive patients with psoriasis vulgaris (PsV) from North-western Poland. Patients were recruited between 2010 and 2012 from outpatient clinics and hospital wards of the two participating dermatology departments: The Chair and Clinic of Skin and Venereal Diseases, PMU, Szczecin; and The Clinic of Dermatology and Venereology, PUMS, Poznań. Participation rates were over 80% for both centres. All patients were at least 18 years old, although the disease could have been diagnosed at an earlier age.

A detailed history of ancestry and clinical data concerning the age of the patient at diagnosis, the family history of psoriasis, smoking habits, personal cancer history and family cancer history was collected.

Controls consisted of 517 healthy adults, who were gender and age-matched case by case. All controls came from the West Pomerania region of Poland. Control samples were selected from a population-based study of 1.5 million individuals from West Pomerania who were enrolled in a study aimed at identifying familial aggregations of malignancies, which was recently performed by our centre. Individuals with psoriasis or a positive family history of psoriasis or cancer were excluded from the control group.

The study conformed to the Declaration of Helsinki, and all participants signed a form of consent prior to the donation of a blood sample. The study was approved by the institutional review board of the Pomeranian Medical University.

## Methods

In the first stage of the study, we compared frequencies of malignant tumour occurrence and the age of the patient at diagnosis in PsV families against those of the general Polish population. We evaluated malignancies affecting probands and their 1^st^ degree relatives.

For statistical analyses, the Chi-Square test, U-test and odds ratio were utilised. Additional analyses performed in PsV families included the comparison of observed frequencies (OF) with expected frequencies (EF) and the relative risk (RR) of occurrence of malignancies at different sites of origin. OF and EF were calculated through evaluation of the total number of family members and affected cases in different age groups (range of 5 years) in PsV families in comparison to age-specific incidence rates in different age groups (range of 5 years) per 100,000 people. These calculations were done by site with individuals registered in the general population of Poland [25]. Bonferroni correction was used for multiple testing.

In the second stage of the study, we compared the prevalence of common alterations in DNA reported in literature to cancer susceptibility alterations among PsV patients, healthy controls and the general Polish population (data obtained from published reports). We genotyped founder BRCA1 mutations (5382insC, C61G, 4153delA), common variants of XPD (D312M, K751Q), NOD2 (3020insC), CDKN2A (A148T), CHEK2 (CHIVS2, I157T), VDR (M1T), p53 (P72R), ATM (E1978X), MC1R (R160W, R151C, R163Q), MTHFR (A222V) and rs67 (rs6983267).

DNA samples were obtained from the peripheral blood of individuals. All SNPs were analysed by real-time PCR, using a LightCycler480 from Roche. The analyses were performed using the TaqMan(R) genotyping assay, which consists of sequence specific primers and oligonucleotide fluorescent labelled probes, enabling amplification of the examined fragments and further allele discrimination. Randomly selected probes were sequenced to confirm the results of real-time PCR.

Power calculations were done *a posteriori* based on the observed versus expected frequencies, the number of individuals analysed and the threshold p-value (after correction for multiple testing by Bonferroni). The significance threshold for the p-value was 0.05/24 for analysis of the genetic markers, and in the case of analysis via the cancer site, the threshold was 0.05/19 for women and 0.05/17 for men (0.05 divided by the amount of hypotheses tested for the same group).

## Results

Evaluation of the tumour spectrum revealed no statistically significant differences between proportions of malignancies observed in our PsV families when compared to the general population (Table 1). Analysis of the mean age of diagnosis (performed in our families for cancers affecting at least 5 cases) also revealed no significant differences between malignancies diagnosed among members of PsV families and the general population.

**Table 1:** Proportion and age at diagnosis of malignancies of different site of origin in PsV families and Polish population

**Tumor site**	**Males from psoriasis families**		**Population (males)#**		**Females from psoriasis families**		**Population (females)#**	
	**Frequency (%)**	**Mean age at diagnosis (yrs)**	**Frequency (%)**	**Mean age at diagnosis (yrs)**	**Frequency (%)**	**Mean age at diagnosis (yrs)**	**Frequency (%)**	**Mean age at diagnosis (yrs)**
Breast	0	ND^	0,2	64,1	17,1	56,8	22,4	60,2
Lungs	24,4	59,4	21,1	65,9	11,1	57,2	8,6	65,1
Colorectal	14,1	60,6	12,4	67,2	7,7	64,8	10,1	68,3
Stomach	4,7	65,6	4,9	66,4	1,7	ND^	2,7	68,6
Prostate	14,1	66,1	13,2	70,1	-	-	-	-
Kidney	3,8	67,5	4,1	63	5,1	63,3	2,8	65,2
Larynx	7,5	56,5	2,7	62,4	0,8	ND	0,4	62,2
Melanoma	1,9	ND	1,7	60,4	4,3	52,6	1,9	58,7
Skin	1,9	ND	6,8	69,3	3,4	ND	7,5	70,2
Leukaemia	4,7	52,6	2,2	56,9	4,3	58,2	1,9	62
Cervix	-	-	-	-	6	58,5	4,4	56,8
Liver	4,7	59,2	1,2	65,8	8,5	63,7	0,8	69,7
CNS	5,7	54,2	2,4	55,3	6,8	61	2,3	59,3
Bladder	1,9	ND	7,0	68,3	0	ND	2,0	68
Pancreas	1,9	ND	2,3	64,7	2,6	ND	2,3	69,2
Uterus	-	-	-	-	2,6	65,6	7,3	63,6
FGT*	-	-	-	-	9,4	48,6*	17,8	68,8*
Hodgkin’s Disease	1,9	ND	0,5	39,6	0	ND	0,5	37
NHLs	0,9	ND	1,4		0,8	ND	1,9	

#- general Polish population, according to data published by National Cancer Registry (Zatoński 2010).

^ND-not done-mean age was calculated for malignancies affecting at least 5 individuals.

*-p<0.05, statistically significant, FGT-female genital tract- site of origin could not be determined more precisely.

**Table 2:** Expected and observed frequencies, related risk of occurrence of malignancies of different site of origin in psoriasis families

**Tumor site**	**Gender**	**Observed cases**	**Expected cases**	**Relative risk (RR, CI 95%), p**
Breast	Females	20	21	1.0, p= 0,8736
Lungs	Males	26	26	1.0, p=1.0
	Females	13	9	1,2, p=0,3891
Colorectal	Males	15	16	1.0, p=0,8553
	Females	9	11	0.9, p=0,6518
Stomach	Males	5	6	0.9, p=0,7618
	Females	4	10	0.6, p=0,1066
Liver	Males	1	1	1.0, p=1.0
	Females	2	1	1.3,p=0,5632
Melanoma	Males	2	2	1.0, p=1.0
	Females	5	2	1.4, p=0,2554
Kidney	Males	4	5	0.9, p=0,7378
	Females	6	4	1.2, p=0,5253
Prostate	Males	15	16	1.0, p=0,8553
Larynx	Males	6	3	1.3, p=0,3152
	Females	1	0,5	1.3, p=0,1964
CNS	Males	3	2	1.2, p=0,6539
	Females	4	2	1.4, p=0,4130
Leukaemia	Males	5	2	1.4, p=0,2552
	Females	5	2	1.4, p=0,2554
Hodgkin Lymphoma	Males	2	0,2	1.8, p=0.002*
	Females	0	0	-
Bones	Males	1	1	1.0, p=1.0
	Females	2	1	1.3, p=0,5632
Bladder	Males	1	1	1.0, p=1.0
	Females	0	2	P=0,1569
NHL	Males	1	1	1.0, p=1.0
	Females	1	1	1.0, p=1.0
Pancreas	Males	2	3	0.8, p=0,6539
	Females	3	3	1.0, p=1.0
Uterus	Females	3	7	0.6, p=0,2039
Ovary	Females	4	5	0.9, p=0,7379
Thyroid	Males	0	0.5	P=0.0523
	Females	2	2	1.0, p=1.0
Cervix	Females	5	4	1.1, p=0,7379
Non-melanoma skin cancer	Males	9	9	1.0, p=1.0
	Females	9	8	1.0, p=0,8069

*p-after Bonferroni correction.

There was a tendency for an earlier appearance of leukaemia (~4 years), laryngeal cancer (~6 years) and melanoma (among males ~6 years) in PsV families (Table 1).

We observed 196 cancers among 3252 members of 517 psoriasis families. In comparison to the expected frequency (190 tumours), the difference was not significant (p=0.7931), neither for males (p=1.0) nor females (p=0.6519).

A statistical comparison of the observed and expected frequencies of cancers revealed a higher than expected occurrence of Hodgkin’s lymphoma among males in PsV families when compared to the general population (OR=1.8, 95%CI 1.6-2.1, p=0.002) (Table 2). There was a tendency, although non-significant, for melanoma overrepresentation among females from PsV families (OR=1.4, p=0.26). A similar tendency for both males and females was observed for leukaemia (OR=1.4, p=0.26) and laryngeal cancer (OR=1.3, p>0.19).

Molecular examination of 17 mutations/polymorphisms in 10 cancer susceptibility genes in psoriasis patients and controls showed no major differences in distribution of cancer susceptibility mutations among PsV families and healthy controls (Table 3).

**Table 3:** Prevalence of the examined mutations/polymorphisms among PsV cases and healthy controls

**Gene (Mutation)**	**Cases**	**Controls**	**p**	**OR**
BRCA1 (ex20) (ex5-300)	2 / 507 0,39%	1) / 510 0,20%	0,5597	2,016
	1 / 507 0,20%	0	0,3156	3,024
CHK2 (I157T)	22 (+) / 503 4,37%	28 (+) / 507 5,52%	0,4000	0,7824
CHK2 (IVS2+1G>A)	2 (+) / 503 0,40%	2 (+) / 507 0,39%	0,9937	1,008
NOD2 (3020insC)	43 (+) / 500 8,60%	41 (+) / 510 8,04%	0,7469	1,076
XPD 936 (D312M) CT TT	200 / 491 40,73%	199 / 493 40,37%	0,9064	1,015
	127 / 491 25,87%	114 / 493 23,12%	0,3173	1,160
XPD 2253 (K751Q) GT GG	240 / 503 47,71%	240 / 510 47,06%	0,8347	1,027
	82 / 503 16,30%	89 / 510 17,45%	0,6255	0,9213
MC1R (R151C) CT TT	33 / 428 7,71%	35 / 501 6,99%	0,6727	1,112
	0 / 428 0%	2 / 501 0,40%	0,1907	0,2331
MC1R (V60L) GT TT	50 / 448 11,16%	63 / 510 12,35%	0,5681	0,8914
	1 / 448 0,22%	6 / 510 1,18%	0,0839	0,1879
MC1R (R163Q) AG AA	30 / 435 6,90%	37 / 501 7,38%	0,7724	0,9289
	1 / 435 0,23%	2 / 501 0,40%	0,6476	0,5749
VDR (M1T) AG AA	240 / 490 48,98%	252 / 508 49,61%	0,8431	0,9752
	99 / 490 20,20%	99 / 508 19,49%	0,7768	1,046
CDKN2A (A148T)	26 (+) / 501 5,19%	28 (+) / 511 5,48%	0,8375	0,9442
p53 (P72R) CG CC	192 / 503 38,17%	195 / 512 38,09%	0,9778	1,004
	34 / 503 6,76%	33 / 512 6,44%	0,8403	1,052
rs6983267 GT TT	261 / 501 52,10%	234 / 509 45,97%	0,0516	1,278
	119 / 501 23,75%	123 / 509 24,17%	0,8779	0,9776
ATM (E198X)	0 (+) / 499 0%	0 (+) / 508 0%	-	-
MTHFR (A222V) CT TT	223 / 488 45,70%	227 / 499 45,49%	0,9483	1,008
	47 / 488 9,63%	44 / 499 8,82%	0,6587	1,102

For analysis of genetic markers, the statistical power *a posteriori* ranges between 0.12 in the most favourable case (marker rs6983267, variant GT) and 0.001 in the least favourable case (marker CHK2, variant IVS2+1G>A). Comparatively, for the most favourable case, the minimum sample size for a statistical power *a priori* of at least 0.7 should have been great than 1800 cases and an equivalent number of controls.

For analysis by cancer site, the statistical power *a posteriori* ranges between 0.17 in the most favourable case (female lung cancer) and 0.0015 in the least favourable case (several types of cancer, e.g. male liver cancer). Comparatively, for the most favourable case, the minimum sample size for a statistical power *a priori* of at least 0.7 should have been greater than 12400 first degree relatives.

## Discussion

Tumour spectrum and the age of the patient at diagnosis of malignancies of different sites of origin observed in our PsV families and in the general population were similar. However, there was a higher than expected frequency of the occurrence of Hodgkin’s lymphoma in males from PsV families. We also observed a tendency towards overrepresentation of leukaemia and laryngeal cancer among PsV families.

Our findings are consistent with the results of a follow up study of Swedish hospitalised psoriasis patients, which point to a significant excess of Hodgkin’s lymphoma and cancers of the upper aerodigestive tract [26]. An increased risk of Hodgkin’s lymphoma and laryngeal cancer has also been reported for Finnish hospitalised patients [18]. The association of leukaemia and psoriasis is not well-documented. There are a series of case reports of leukaemia developing in psoriasis patients treated with immunosuppressive drugs, such as cyclosporine, methotrexate or etanercept [27-31]. Our results support the theory that psoriasis is not associated with an increased risk of NHL overall or of any NHL subtype [32], although there are some reports suggesting that such an association exists [16,18]. We observed a non-significant tendency of melanoma overrepresentation among females from PsV families. This insignificant correlation does not clarify the inconsistent data from literature regarding the association of melanoma with psoriasis. We show that the risk of melanoma is not significantly increased for individuals from PsV families.

Due to the relatively small numbers of cases, both type 1 and 2 statistical errors cannot be excluded; the results thus need to be verified by examination of a larger series of patients. The current study lacks the statistical power to confirm if the absence of observed association is due to a true lack of association or rather due to an insufficient sample size. The current research should be considered only as an exploratory study attempting to reveal interesting tendencies worthy of in-depth analysis at a later stage.

Assessing the risk of cancer as a single outcome revealed no statistically significant association between psoriasis and cancer. It indicates that the common tumours (such as cancers of the breast, prostate, lung or colon) constituting the majority of neoplasms are not strongly linked with psoriasis. However, it does not exclude a moderate association for rare malignancies.

The results of molecular genotyping of mutations/polymorphisms present in cancer susceptibility genes revealed no differences in the prevalence of the examined alterations between cases and controls. It seems that none of the examined alterations in DNA are associated with psoriasis. This is the first such study evaluating any linkage between the BRCA1 gene (predisposing to breast and ovarian cancer), CHEK2 gene (cancers of the prostate, thyroid, breast, colon, stomach), MC1R gene (melanoma, non-melanoma skin cancers), ATM gene (cancers of the breast, pancreas, thyroid) and the XPD gene (melanoma, oesophageal squamous cell carcinoma, cancers of the lung and breast) with psoriasis. Our results are consistent with current data in literature, suggesting that NOD2 (colorectal and breast cancer), p53 (cancers of the cervix, bladder, prostate, breast) and MTHFR (oesophageal and cervical cancers) might not be susceptibility genes for psoriasis [33-36]. Our findings support the thesis that the VDR gene (predisposing to melanoma and breast cancer) does not show a robust and reproducible association with the risk of psoriasis [37]. Since this theory conflicts with other findings [22,23,38], any association that may exist is likely to be weak and potentially restricted to specific populations. Down-expression of another cancer susceptibility gene, CDKN2A (predisposing to melanoma, cancers of the pancreas, breast and lung), led to speculation that the gene may have a role in the pathogenesis of psoriasis [24]. Our findings do not support this thesis.

According to many follow-up studies, there is an increased risk of the most common human malignancy, basal cell cancer (BCC), among psoriasis patients [10-13]. In a recent study, genetic susceptibility to basal cell carcinoma (BCC) among Danish psoriatic patients was investigated and confirmed previous reports that XPD may predispose to BCC [39-41]. Interestingly, we did not observe any excess of non-melanoma skin cancers in our families and found no association of XPD common variants with psoriasis. This lack of association suggests that the risk is restricted to individuals treated with phototherapy, which constituted only a small proportion of the individuals in our PsV families.

In conclusion, the results of our study indicate a moderate association between psoriasis and familial cancer risk. These results also suggest an increased risk of Hodgkin’s lymphoma for male members of PsV families. Further studies are needed to confirm the findings and to evaluate whether or not cancer surveillance protocols are justified in these families.

## Competing interests

Authors declare that they have no competing interests.

## Authors’ contributions

Conception and design: TD, RM. Acquiring, analysis and interpretation of data: TD, ES, MB, MR, JW, AM, KPS, LK, ZA; drafting the manuscript: TD, PSF, RM; critical revision for important intelectual content: TD, RM; final approval: TD,ES, MB, MR, JW, AM, KPS, JL, LK, ZA, RM. All authors read and approved the final manuscript.
